# People’s Financial Choice Depends on their Previous Task Success or Failure

**DOI:** 10.3389/fpsyg.2015.01730

**Published:** 2015-11-17

**Authors:** Katarzyna Sekścińska

**Affiliations:** Faculty of Psychology, University of WarsawWarsaw, Poland

**Keywords:** experience, success, failure, financial choices, saving, invest, risk-taking

## Abstract

Existing knowledge about the impact of the experience prior to financial choices has been limited almost exclusively to single risky choices. Moreover, the results obtained in these studies have not been entirely consistent. For example, some studies suggested that the experience of success makes people more willing to take a risk, while other studies led to the opposite conclusions. The results of the two experimental studies presented in this paper provide evidence for the hypothesis that the experience of success or failure influences people’s financial choices, but the effect of the success or failure depends on the type of task (financial and non-financial) preceding a financial decision. The experience of success in financial tasks increased participants’ tendency to invest and make risky investment choices, while it also made them less prone to save. On the other hand, the experience of failure heightened the amount of money that participants decided to save, and lowered their tendency to invest and make risky investment choices. However, the effects of the experience of success or failure in non-financial tasks were exactly the opposite. The presented studies indicated the role of the specific circumstances in which the individual gains the experience as a possible way to explain the discrepancies in the results of studies on the relationship between the experience prior to financial choice with a tendency to take risks.

## Introduction

Traditional economic theories describe a decision maker as a rational person whose choices reflect an estimation of relevant probabilities and outcomes. Every decision is aimed at maximizing expected value or utility (von Neumann and Morgenstern, 1944). The most important research and theory that changed the way of thinking about decision makers was the prospect theory (Kahneman and Tversky, 1979). Moreover, psychologists emphasize that every decision (including a financial one), when seen in retrospect as good or bad, can affect a person’s mood, self-esteem, and risk preferences, for instance (e.g., Isen and Simmonds, 1978; Josephs et al., 1992). Thus, every choice people make builds their experience, which in turn can determine their subsequent decisions.

### Experience of Success or Failure and Risky Choices

Recent work has suggested that there is a significant relationship between good or bad experiences and risk preferences. The experience of success leads men to take more risks (Lam and Ozorio, 2013). Moreover, people who have experienced success are more likely to make risky choices than those who have experienced failure (Zawadzka and van Buuren, 2010). A study by Ludvig et al. (2015) showed that evoking memories of wins makes people more prone to risky choices.

The relationship between the experience of failure and risk preferences seems to be different. Lam and Ozorio (2013) showed that failure tends to make women take more risks. Other research indicated that the experience of serious financial loss (e.g., as a result of hyperinflation) lowers people’s risk-taking (Klement and Miranda, 2012). Bad financial decisions in business made by people with little experience lowers their risk-taking self-efficacies (Forlani, 2013).

The experience of success can raise a person’s self-esteem and self-affirmation (Josephs et al., 1992). It can make people feel more competent in the field of the task (e.g., Bandura and Cervone, 1986). They can also become overly optimistic (in the case of financial tasks ignoring the principle of regression to the mean), more confident in their predictions, less sensitive to objective indicators of the situation and more susceptible to illusions of control (e.g., Bandura and Cervone, 1986). Finally, after the experience of success in some task people may prefer to undertake other, similar activities. Failure, however, creates the opposite effect. Both the experience of success and the experience of failure can cause mood changes (Isen and Simmonds, 1978). The experience of success may put people in a good mood, while the experience of failure during a bad mood that can influence their further decisions.

### Mood and Risk

Past research has indicated various mechanisms that mediate the relationship between mood and risk preferences. Mood maintenance theory states that people who are in a good mood seek to maintain the status quo, therefore prefer safer alternatives (Mischel et al., 1973; Isen et al., 1988; Leith and Baumeister, 1996; Nygren et al., 1996).

Another mechanism is described in the affect infusion model (AIM) that explains the role of affective states in people’s judgments and thinking (Forgas, 1995). The model explains how people select, learn and interpret new information about a situation and incorporate them into the knowledge they already have. Under this theory, the more people need to engage in constructive processing, the more likely that their affective state will indirectly or directly influence their judgments (Forgas, 1995). According to the AIM, a good mood makes people willing to take a risk, if they have experienced positive outcomes (respectively good mood) from making risky decisions.

Mood congruence hypothesis refers to the influence of mood on memory. Under this hypothesis memories associated with certain emotions can be recalled by similar emotions (Au et al., 2003). Therefore, people in a good mood recall good rather than bad memories. But the role of good and bad mood is not the same. Good mood makes people more prone to recall good memories and less prone to recall bad memories, bad mood decreases people’s propensity to recall good memories, but does not increase their propensity to recall bad memories (Weiss and Cropanzano, 1996).

Mood has also been shown to affect the type of cognitive processing strategies utilized. People in a good mood try to simplify search procedures and prefer a more heuristic path while processing information (Au et al., 2003) resulting in a variety of cognitive biases (Wyer et al., 1999). For instance, people in a good mood rely on stereotypes (Bodenhausen, 1993), use only a few categories to classify a new object (Isen and Daubman, 1984) and spend less time deciding (Forgas, 1989). People in a pleasant mood are more optimistic, confident and affected by illusions of control (Au et al., 2003). People in a bad mood process information differently. They prefer more algorithmic ways, making decisions with greater accuracy and reduced variety of biases (Taylor and Brown, 1988; Sinclair and Mark, 1992). People in a bad mood are also less affected by illusions of control than people in a good mood (Alloy and Abramson, 1988).

Subjective probability weighting is another factor that can mediate the relationship between mood and risk taking. People in a good mood estimate higher probability of positive outcomes than people in a bad mood. They also assess bad results as less likely than people in a bad mood (Wright and Bower, 1992). Moreover, research by Nygren et al. (1996) showed that people in a positive mood underestimate the probabilities of negative events and overestimate the probabilities of positive ones.

There are numerous studies that have shown the role of mood on risk preferences. Some studies indicated that positive mood is connected with risk avoidance, in accordance with the mood maintenance hypothesis (Mischel et al., 1973; Isen et al., 1988; Leith and Baumeister, 1996; Nygren et al., 1996). However, other studies indicated that a positive mood is connected with risk-taking (Nygren et al., 1996; Isen, 1997; Mittal and Ross, 1998), in accordance with the other theories described above. On the other hand, some prior studies showed that a negative mood lowers people’s tendency to make risky decisions (Yuen and Lee, 2003), but for example a study conducted by Bruyneel et al. (2009) showed that a negative mood can sometimes increase the propensity for risky decisions if the person has enough time to actively regulate his or her mood before making the decision. Other research has indicated, moreover, that the effort to reduce a negative mood can result in risky behaviors (Tice et al., 2001; Andrade, 2005).

The results of studies on the impact of mood on risky choices are not consistent. However, an attempt to explain the discrepancies has already been made. Isen and Geva’s (1987) studies showed that people in a good mood prefer lower risk when the loss is meaningful and possibility of loss is high, but they are prone to take more risk when the consequences of choice are trivial and the chance of losing is low. According to the contingent theory of risk – taking (Au et al., 2003), the role of mood depends on the specifics of the risk-task. If the task is well-defined, special skills are not needed to make a good choice and the probabilities of gain and loss are known, then mood maintenance is the prime motive for behavior (Au et al., 2003). Thus, a good mood leads to more risky choices. But, when the situation is ambitious, not well known, and probabilities of gain and loss are not given, then the illusion of control, optimism, overestimation of the probability of success and underestimation of the probability of failure are crucial in decision-making (Au et al., 2003). Therefore, in ambitious situations good mood encourages risk-taking while bad mood makes people more risk–averse.

### The Present Studies

The experience of success and failure causes mood changes that may influence the effects of experience on people’s risky choices. In the literature one can find attempts to explain inconsistencies in results on mood and risk that indicate the role of the specifics of the task that involves a potentially risky choice, including the level of difficulty of the task, familiarity with specific tasks, subjective assessment of significance of the results, etc. (Isen and Geva, 1987; Au et al., 2003). All explanations focus on the specific characteristics of decisive situation, which does not seem to fully answer the question about the causes of observed non-compliance results of the relationship between mood and risk-taking.

It seems worthwhile paying attention not only to the task of risky choice, but also for the task in which the experience of success or failure prior to the decision was gained, which is a potential source of mood changes. It can be expected that the experience of success/failure in the task similar to further decision situations (e.g., experience in a financial task prior to investment decision), can affect the mood of the individual, but also her or his assessment of own competence, confidence of predictions and may increase errors associated with assessment of the decisive situation, e.g., estimation of the probabilities of a particular results (Bandura and Cervone, 1986; Forlani, 2013). While the influence of experience in a situation unrelated to the further task (e.g., financial choice preceded by coloring pictures) may be different. Undoubtedly, it can still affect mood (Isen and Simmonds, 1978), but probably has a much lower impact on the assessment of one’s own skills and competence, confidence of one’s own predictions in a completely different context, and assessment of different outcome probabilities.

The starting point for deliberations on consumption and saving is very often consumption function presented in Keynes (1936/2014). The amount that households spend on consumption depends “*partly on the amount of its income, partly on other objective attendant circumstances, and partly on the subjective needs and the psychological propensities and habits of the individuals composing it and the principles on which the income is divided between them (ibidem. Chapt. 8).”* Keynes concluded that real disposable income is the most important determinant of consumption and saving. He put forward a psychological law of consumption, according to which, as income increases consumption increases but not by as much as the increase in income (ibidem). In other words, marginal propensity to consume is more than zero but less than one. In Keynes’ theory the average propensity to consume (APC) decreases with increasing income (ibidem). Thus the greater the disposable income, the higher the part of the income allocated for savings should be. The early empirical findings supported Keynes’ conjectures about consumption (e.g., Hall and Taylor, 1993). But in the 1940s empirical studies of long-term times series data from the US economy for the period 1869–1938 by Simon Kuznets showed that consumption was in fact stable in spite of rising income and APC was relatively stable over long periods. Further studies confirmed Kuznets’ findings (e.g., Snowdown and Vane, 2005). Evidence therefore indicated that there are two different consumption functions: a short term consumption function with the variable value of the APC and a long-run consumption function in which the APC was constant regardless of the level of income. A short term consumption function seemed to confirm Keynes’ conjectures. According to Keynes’ theory in the short term people differently manage a little than a large amount of disposable money (Samuelson and Nordhaus, 1998), therefore in the current studies I tested financial choices in three situations, where each person has a small, medium and large amount of disposable money.

Previous studies on the role of the experience of success or failure have focused on explaining risky decision-making, though only a few of them have addressed financial choices. Moreover, most of them utilized the gamble task that offered a 50% chance of winning and 50% chance of losing some money, which is not very similar to people’s every day financial choices. In addition, there was no distinction between the propensity to invest and risky investment choices in the previous studies. This distinction seems to be important because people may want to invest, but safely. In addition, previous studies related to the financial choices concerned only gamble games, lotteries and risky investment choices. Actual people’s choices, however, include more categories. In everyday life, people choose between consumption, saving and investing.

A justified question in the presented studies is whether the impact of the experience of success or failure on the financial choices is different if the experience comes from specific tasks in the context of money, than when it was gained in a non-specific task (not associated with money). Moreover, the studies presented in this paper were intended to complement existing research on the effects of experience and mood on financial behavior, indicating the impact of these variables on daily financial choices, including propensity to consume, save and invest and willingness to build risky investment portfolios. It was hypothesized that experience of success or failure in prior financial and non-financial tasks would influence people’s financial choices, especially their tendency to consume, save, invest (Hypothesis 1), and to build risky investment portfolios (Hypothesis 2). It was also hypothesized that the role of the experience on financial choices may be different when it comes from specific (financial) that non-specific (non-financial) tasks (Hypothesis 3).

## Study 1 – How the Experience of Financial Tasks Affects Financial Choices

The main aim of this study was to examine whether and how the experience of success or failure in prior financial tasks affects peoples’ tendency to consume, save, invest and make risky investment choices.

### Participants

In order to increase the reliability of the study, the chosen participants were financially independent adults with their own income, therefore having the potential to gain future investing experience. They were 259 entrepreneurs and sales workers from four Polish cities of different sizes. The participants’ ages ranged from 19 to 67 years (mean = 35.18, *SD* = 10.4), and 79% were women. The Ethics Board of the Faculty of Psychology at the University of Warsaw approved the study, which was carried out in accordance with the Board’s recommendations. At the end of the study, the participants were fully debriefed.

### Method

The study was conducted with three groups: two experimental and one control. Participants were randomly assigned to each group, in which neutral financial experience (control group, *n* = 87) or an experience of financial failure (failure group, *n* = 86) or success (success group, *n* = 86) was created. The participants were shown 10 charts presenting different share pricing, and were asked to choose which one or ones they believed would generate the highest profits within half a year. The participants were informed that charts presented actual historical data of 10 companies listed on the Warsaw stock exchange.

After the participants had made their choices, the experimenter told them that he knew more recent prices of individual stocks and could say what financial consequences the participants’ choices would cause. The participants of the first experimental group (success group) were then told that their choices were very good and would bring them money; they therefore experienced success. The participants of the second group (failure group) were told that their choices were very bad and would incur a loss, so they experienced failure. The control group participants did not receive any feedback. The type of experience (success, failure, control) was a between subjects IV. The effectiveness of manipulation was measured in the pilot studies. After completing the task and obtaining feedback, participants were asked to answer the question: “How do you rate your present mood?” They rated their mood on a scale 0 (very bad) to 10 (very good). The group that experienced failure assessed their mood significantly lower than the group who experienced success (*N* = 72; *t*[66] = 2.145, *p* < 0.05, *Cohen’s d* = 0.51).

After an experimental manipulation, the participants were told what consuming, saving, and investing meant in the context of the study. Consuming meant spending money on hand on products or services. Saving meant keeping the money in non-profitable (or almost non-profitable) form, without the risk of loss, e.g., deposit in a non-interest-bearing bank account. Investing was defined as allocating funds to financial instruments that can generate profits but with the risk of losses, e.g., allotment to stocks or mutual funds.

Then participants were asked to make various hypothetical financial decisions. Hypothetical choices could be argued as a limitation of the present studies. Some decision experiments showed that real monetary reward are stronger incentives than nominally equivalent hypothetical reward (Camerer and Hogarth, 1999; Hertwig and Ortmann, 2001). However, studies directly comparing large real reward with large hypothetical reward have provided some evidence that the results of experiments with hypothetical reward validly apply in everyday life. Kuhberger et al. (2002) showed no differences in financial choices between participants who imagined a hypothetical gamble and those gambling for real money. Other researchers found in their studies that discounting rates for real and hypothetical reward did not differ significantly (Johnson and Bickel, 2002; Locey et al., 2011).

The first task aimed to capture people’s propensity for different financial choices indicated by the money amount assigned by participants to different categories (DV). The participants were asked to distribute a hypothetical amount of money^1^ between consumption, savings, and investment (within subjects IV). This amount was small (500 PLN; ∼135 USD), medium (3000 PLN; ∼800 USD) and large (10,000 PLN; ∼2655 USD). Participants in all three groups were asked to dispose of all three sums of money (level of money was a within subjects IV).

Finally, the participants’ propensity to take investment risks was assessed. All participants were asked to create an investment portfolio by indicating what percentage of the hypothetical amount of money (10,000 PLN; ∼2655 USD) they wanted to allocate to a variety of financial market instruments (DV). The participants could invest their hypothetical money in bonds, balanced mutual funds (that invest 50% in stock and 50% in bonds) and stocks (within subjects IV). The participants had the opportunity to select one or more of the instruments mentioned above (e.g., they could allocate the entire sum of money to stocks or divide it between two or more categories of investment instruments). The objective of this task was to check the participants’ propensity to invest in bonds, mutual funds and shares (indicated by the percentage of amount allocated to these investment instruments). The task was also to check general riskiness of the created portfolio. The indicator of the overall riskiness of the created portfolio reflected the percentage of shares (instruments that are affected by significant risk of loss) in the portfolio (DV). The indicator was based on the formula: 0 × percentage of bond + 0.5 × percentage of fund + 1 × percentage of the shares (therefore 0 was the lowest possible value of the indicator, meaning the safest portfolio, and 100 was the highest possible value of the indicator, meaning the riskiest portfolio).

All the tools used in the study were developed by the author of this paper, and all were checked in a several previous pilot studies.

### Results

#### Effects of Success or Failure on Propensity to Consume, Save and Invest

To test the first research hypothesis, a 3 (experience: success, failure, control – between subjects IV) by 3 (amount levels: small, medium, large – first within subjects IV) by 3 (way of spending money: consumption, savings, and investment – second within subjects IV) mixed-design analysis of variance (ANOVA), with amount of money assigned by participants as a DV, was conducted. A significant interaction between experience, way of spending money and amount levels was obtained (*F*[8,1024] = 3.506, *p* < 0.05, η^2^ = 0.027). This suggested that money assigned by the participants to each way of spending money differed depending on both type of experience and level of divided money. The interaction between experience and way of spending money was also significant (*F*[4,512] = 5.945, *p* < 0.001, η^2^ = 0.044). The amount allocated for consumption in all groups was similar, but participants in the failure group and the control group were willing to spend more money on savings than investment, whereas in the success group the relationship was reversed.

**FIGURE 1:**
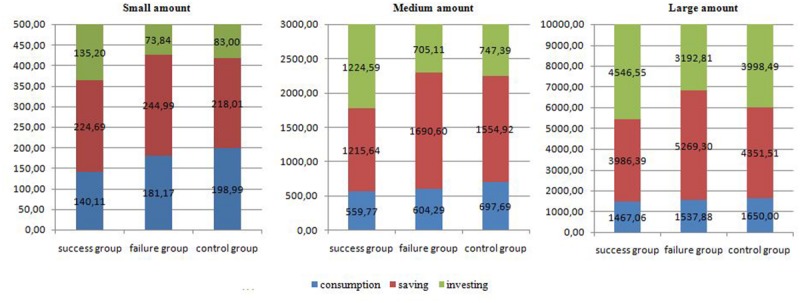
**Money assigned to consumption, saving, and investing by each experience group for different amount levels (Mean amount in PLN) – Study 1**.

In order to perform the follow-up tests, the data set was split according to the levels of the variables involved in the significant interaction between experience, way of spending money and amount levels. In a first step, the set was divided into the three amount levels (small, medium, large). Three 3 (experience: success, failure, control – between subjects IV) by 3 (way of spending money: consumption, savings, and investment–within subjects IV) mixed-design ANOVAs were conducted. The interaction between experience and way of spending money was significant for small (*F*[4,512] = 2.648, *p* < 0.05, η^2^ = 0.020), medium (*F*[4,512] = 8.148, *p* < 0.001, η^2^ = 0.060) and large (*F*[4,512] = 4.267, *p* < 0.05, η^2^ = 0.032) money level. The means (**Figure 1**) showed that for the small amount, the largest money was assigned to consumption after neutral experience (control group) and the smallest after success. A larger amount was spent on saving if participants experienced failure, while an amount assigned to invest was larger after success. When the amount level was medium a higher propensity to consume was observed after neutral experience. People decided to assign the highest amount to savings after the experience of failure and the smallest after success. A higher part of money was spent on investments if participants were from the success group. For the large amount level, propensity to consume was similar for all experience groups. However, the money assigned to savings was the highest when the failure group decided and the smallest when participants from the success group made a decision. For the investments the pattern was the opposite.

To correctly interpret the obtained interactions and to check the relationships presented above, the small, medium, and large amount subsets were divided according to the three categories of spending money *(*consumption, savings, and investment) and ANOVAs were run to compare each experience category (success, failure, neutral). The results are listed below, by category of spending money.

##### Effects of success or failure on consumption

The results of the ANOVA showed that for the small amount of money divided, the difference between the three analyzed groups in amount spent on consumption was significant (*F*[2,256] = 3.161, *p* < 0.05, η^2^ = 0.024). Furthermore, *t*-tests showed that participants from the success group were prone to spend less money on consumption than the control group (*t*[165] = 2.328, *p* < 0.05, *Cohen’s d* = 0.35). The ANOVA showed no statistically significant differences between the three conditions for medium (*F*[2,256] = 1.717, *p* = 0.18, η^2^ = 0.013) and large (*F*[2,256] = 0.467, *p* = 0.63, η^2^ = 0.004) amount of money divided. The descriptive statistics are presented in **Figure 1**.

##### Effects of success or failure on saving

With a one-way ANOVA, the amount spent on saving for the success, failure and control groups were compared. The descriptive statistics are presented in **Figure 1**. There were no significant differences observed when the divided amount was small (*F*[2,256] = 0.660, *p* = 0.52, η^2^ = 0.005). For the medium amount, ANOVA indicated that the difference between the groups was significant (*F*[2,256] = 7.824, *p* < 0.001, η^2^ = 0.058), with the success group’s propensity to save being lower than that of the control (*t*[170] = 2.342, *p* < 0.05, *Cohen’s d* = 0.36) and failure (*t*[166] = 4.107, *p* < 0.05, *Cohen’s d* = 0.62) groups. Moreover, a significant manipulation effect was observed for the propensity for saving a large amount (*F*[2,256] = 4.665, *p* < 0.05, η^2^ = 0.035). Further t-tests showed that participants that experienced failure tended to save more than those from the success (*t*[256] = 2.926, *p* < 0.05, *Cohen’s d* = 0.45) or control (*t*[256] = 2.226, *p* < 0.05, *Cohen’s d* = 0.34) group.

##### Effects of success or failure on investing

There were significant differences in the amount assigned to investments between the experience groups in all three levels of amount: small 500 PLN – *F*(2,256) = 5.206, *p* < 0.001, η^2^ = 0.039; medium 3000 PLN –*F*(2,256) = 10.136, *p* < 0.001; η^2^ = 0.073; large 10 000 PLN – *F*(2,256) = 4.619, *p* < 0.05, η^2^ = 0.035). The contrast tests showed that the participants from the success group who were assigned the small and medium amounts were more prone to invest than those from the failure (small amount: *t*[143] = 3.113, *p* < 0.05, *Cohen’s d* = 0.48; medium amount: *t*[165] = 4.009, *p* < 0.001, *Cohen’s d* = 0.61) and control (small amount: *t*[170] = 2.289, *p* < 0.05, *Cohen’s d* = 0.35; medium amount: *t*[170] = 3.383, *p* < 0.001, *Cohen’s d* = 0.54) groups. Furthermore, the amount spent on investment was higher in the success group than in the failure group, when the large amount of money was divided (*t*[256] = 3.035, *p* < 0.05, *Cohen’s d* = 0.48). The mean values are presented in **Figure 1**.

#### Effects of Success or Failure on Risky Investment Choices

To verify the second research hypothesis an ANOVA test revealed differences between the three experimental groups (success, failure, control) in terms of the risk level of the created investment portfolios (*F*[2,256] = 4.967, *p* < 0.05, η^2^ = 0.037). The means for each group were: success group = 48.61, failure group = 35.87, control group = 38.38. Further analysis showed that the success group built riskier portfolios than both failure (*t*[256] = 2.945, *p* < 0.05, *Cohen’s d* = 0.44) and control (*t*[256] = 2.472, *p* < 0.05, *Cohen’s d* = 0.37) groups.

**FIGURE 2:**
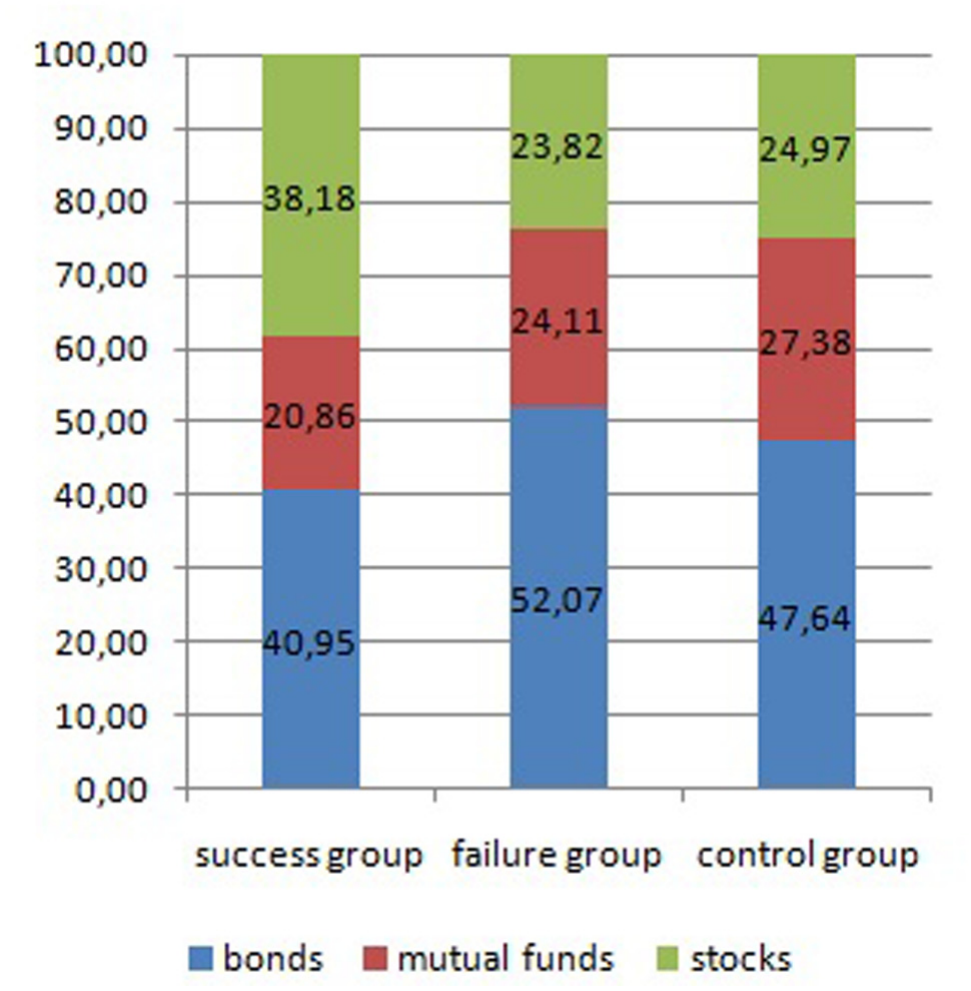
**Percentage of money assigned to invest in different forms of instruments in success, failure, and control groups – Study 1**.

Further analysis aimed to check whether the experimental groups differed in the money assigned to different forms of investments: bonds, shares, and mutual funds. A 3 (experience: success, failure, control – between subjects IV) by 3 (form of investment: bonds, mutual funds, stocks – within subjects IV) mixed-design ANOVA, with a percentage of amount of money assigned by participants as a DV, was conducted. A significant interaction between experience and form of investment was obtained (*F*[4,512] = 3.573, *p* < 0.05, η^2^ = 0.027). Further ANOVA analysis showed that there were significant differences between the groups in terms of their tendency to invest in bonds (*F*[2,256] = 3.413, *p* < 0.05, η^2^ = 0.029) and shares (*F*[2,256] = 6.555, *p* < 0.001, η^2^ = 0.049). There were no significant differences observed in the amount of money spent on mutual funds (*F*[2,256] = 1.287, *p* = 0.28, η^2^ = 0.010). Further *t*-tests revealed that the success group allocated less money to bonds than the failure group (*t*[256] = 2.280, *p* < 0.05, *Cohen’s d* = 0.35), and more money to stocks than both the failure (*t*[256] = 3.187, *p* < 0.05, *Cohen’s d* = 0.47) and control (*t*[256] = 2.953, *p* < 0.05, *Cohen’s d* = 0.44) groups. The descriptive statistics for each of the groups analyzed are presented in **Figure 2**.

## Study 2 – How the Experience of Non-Financial Tasks Affects Financial Choices

The goal of the next study was to examine whether the influence of failure or success on financial risk observed in the first experiment would only apply to financial failure or success, or if the phenomenon is of a more general nature. Therefore, Study 2 involved observing whether failure or success in non-financial tasks could have the same effect on financial choices.

### Participants

Analogously to Study 1, in order to increase the reliability of the study, the participants in Study 2 were financially independent adults with their own income and the potential to have experience investing money. They were entrepreneurs and sale workers recruited from four Polish cities of different sizes, and aged 18 to 68 years (mean = 36.52, *SD* = 11.8). Of the 271 participants, 75% were women. All the participants gave their informed consent in accordance with the APA Ethical Principles of Psychologists and Code of Conduct. The Ethics Board of the Faculty of Psychology at the University of Warsaw approved the study. The participants were fully debriefed at the end of the study.

### Method

Study 2 also involved three groups—two experimental and one control—to which participants were randomly assigned (success group *n* = 90, failure group *n* = 90, control group *n* = 91). The participants were shown a board of 24 squares, each with the same pattern of five dots (**Supplementary Figure S1**). They were asked to draw a line connecting at least two dots in each square, each in a unique way. The participants had 30 s to complete the task. The participants of the first experimental group (success group) were informed that they had done the task well; i.e., they experienced success. The participants of the second group (failure group) were told that they had done the task poorly: they experienced failure. The participants of the control group did not receive any feedback. The effectiveness of manipulation was measured in the pilot studies in the same way as the manipulation in the first study. The group that experienced failure assessed their mood significantly lower than the group who experienced success (*N* = 70; *t*[66] = 3.316, *p* < 0.05, *Cohen’s d* = 0.79).

Then the participants answered a few questions (the same as the ones used in Study 1) about various hypothetical financial decisions. First, all participants were asked to divide a small (500 PLN; ∼135 USD), medium (3000 PLN; ∼800 USD) and large (10,000 PLN; ∼2665 USD) amount of money between consumption, saving, and investment to verify the participants’ propensity to consume, save and invest. In order to check their propensity for investment risk, they were asked to create an investment portfolio by indicating the percentage of the sum that they wanted to spend on bonds, mutual funds, and stocks. The participants had the opportunity to select any number of the instruments mentioned.

The measured variables in Study 2 were the same as Study 1: between subjects IV: experience (success, failure, control); within subjects IVs: level of amount (small, medium, large), way of spending money (consumption, saving, investing), form of investment (bonds, mutual funds, stocks); DVs: money assigned by participants, percentage of money assigned by participants.

### Results

#### Effects of Success or Failure on Propensity to Consume, Save and Invest

To test the first research hypothesis in a context of experience in a non-financial task, a 3 (experience: success, failure, neutral) by 3 (amount levels: small, medium, large) by 3 (way of spending money: consumption, savings, and investment) mixed-design ANOVA, with an amount of money assigned by participants as a DV, was conducted.

There was a significant interaction between experience, way of spending money and amount levels (*F*[8,1052] = 2.561, *p* < 0.05, η^2^ = 0.019). This result suggested that money assigned by the participants to each way of spending money differed depending on both type of experience and level of divided money. The significant interaction between experience and way of spending money was also obtained (*F*[4,526] = 3.598, *p* < 0.05, η^2^ = 0.027). On the basis of the means it may be noted that in all groups the amount allocated for consumption was the smallest and for investment the largest. But the ratio of the amount allocated to the various categories of way of spending money differed between experience groups.

**FIGURE 3:**
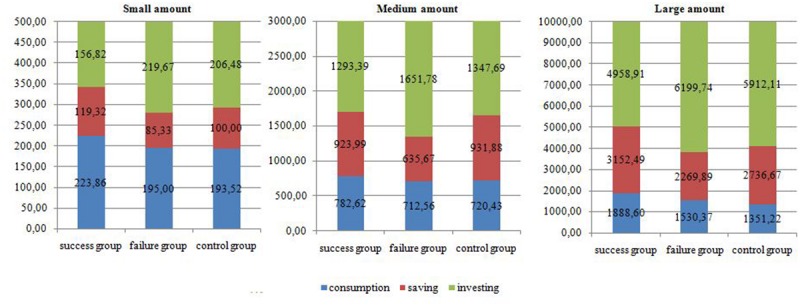
**Money assigned to consumption, saving and investing by each experience group for different amount levels (Mean amount in PLN) – Study 2**.

For further analysis, the data set was split according to the levels of the variables involved in the significant interaction between experience, way of spending money and amount levels. In a first step, the set was divided into the three amount levels subsets (small, medium, large). Three 3 (experience: success, failure, neutral – between subjects IV) by 3 (way of spending money: consumption, savings, and investment–within subjects IV) mixed-design ANOVAs were conducted. The interaction between experience and way of spending money was significant for all levels of money: small (*F*[4,530] = 3.011, *p* < 0.05, η^2^ = 0.020), medium (*F*[4,530] = 3.016, *p* < 0.05, η^2^ = 0.022) and large (*F*[4,528] = 3.030, *p* < 0.05, η^2^ = 0.022) money level. Based on means (**Figure 3**) the small level of amount of money assigned to consumption was larger for the success group than others, money spent on savings were the biggest for the success group and the smallest for the failure group. For the investments the findings were exactly the opposite. When the level of amount was medium, money assigned to consumption was similar for all experience groups. Participants who experienced failure spent less money on savings than others. People were prone to spend the largest amount of money if they experienced failure and the smallest amount after success experience. Finally, for the large level of amount, a higher amount of money was assigned to consumption by participants that experienced success. People were prone to assign the highest amount of money on savings after the experience of success and the lowest amount after the failure. For the money allocated to investments the findings were the opposite.

In order to correctly interpret the obtained interactions and verify the significance of differences in means presented above and, the small, medium and large amount subsets were divided according to the three categories of spending money *(*consumption, savings, and investment) and ANOVAs were run to compare each experience category (success, failure, neutral). The analyses are listed below, by category of spending money.

##### Effects of success or failure on consumption

A one-way ANOVA was used to examine the differences between the three experience groups in the amount of money assigned to consumption (means are presented in **Figure 3**). No significant differences were observed when people were asked to divide the small (*F*[2,269] = 0.997, *p* = 0.37, η^2^ = 0.007) and medium (*F*[2,269] = 0.120, *p* = 0.88, η^2^ = 0.001) amounts of money. However, the ANOVA test indicated differences between the groups when distributing the large amount (*F*[2,264] = 2.528, *p* < 0.005, η^2^ = 0.019). Further *t*-tests showed that the success group tended to spend more money on consumption than the control group (*t*[264] = 2.205, *p* < 0.05, *Cohen’s d* = 0.32).

##### Effects of success or failure on saving

An ANOVA test showed no difference between the groups in terms of the sum of money they assigned to savings when the level of divided amount was small (*F*[2,269] = 1.431, *p* = 0.24, η^2^ = 0.011) or large (*F*[2,269] = 2.240, *p* = 0.11, *η*^2^ = 0.017). Significant differences were observed only when participants were asked to dispose a medium amount (*F*[2,265] = 3.297, *p* < 0.05, η^2^ = 0.024). Further analysis showed that the failure group was prone to save less money than the other experience groups (success vs. failure: *t*[264] = 2.005, *p* < 0.05, *Cohen’s d* = 0.36; success vs. control: *t*[264] = 2.373, *p* < 0.05, *Cohen’s d* = 0.34).

##### Effects of success or failure on investing

The effects of the experience of success or failure on people’s propensity to invest were analyzed using an ANOVA test; the mean values for the experimental groups are presented in **Figure 3**. The results showed statistically significant differences between three experience groups (success, failure and control) in the sums spent on investments for the small (*F*[2,266] = 3.495, *p* < 0.05, η^2^ = 0.026), medium (*F*[2,265] = 3.401, *p* < 0.05, η^2^ = 0.025) and large (*F*[2,264] = 3.857, *p* < 0.05, η^2^ = 0.028) amount levels. Further analysis indicated that the success group was prone to invest less money than the failure group regardless of the amount (small: *t*[168] = 2.584, *p* < 0.05, *Cohen’s d* = 0.31, medium: *t*[265] = 2.481, *p* < 0.05, *Cohen’s d* = 0.36 and large: *t*[264] = 2.642, *p* < 0.05, *Cohen’s d* = 0.39) and less money than the control group when the amount was small (*t*[172] = 2.090, *p* < 0.05, *Cohen’s d* = 0.38) or large (*t*[264] = 2.090, *p* < 0.05, *Cohen’s d* = 0.31). The control group, on the other hand, invested a smaller portion of the medium amount than the failure group (*t*[264] = 2.004, *p* < 0.05, *Cohen’s d* = 0.30).

#### Effects of Success or Failure on Risky Investment Choices

One-way ANOVA was used to examine the differences between all experience groups (success, failure, control) in terms of the risk level of the created investment portfolios. The means for each group were: success group = 34.28, failure group = 42.36, control group = 41.45. The riskiness of the investment portfolios, expressed as a total percentage of stocks in portfolio, differed significantly between the groups (*F*[2,261] = 3.388, *p* < 0.05, η^2^ = 0.158). Contrast tests showed that the success group created safer portfolios than both the failure (*t*[261] = 2.222, *p* < 0.05, *Cohen’s d* = 0.35) and control (*t* [261] = 2.005, *p* < 0.05, *Cohen’s d* = 0.30) groups.

**FIGURE 4:**
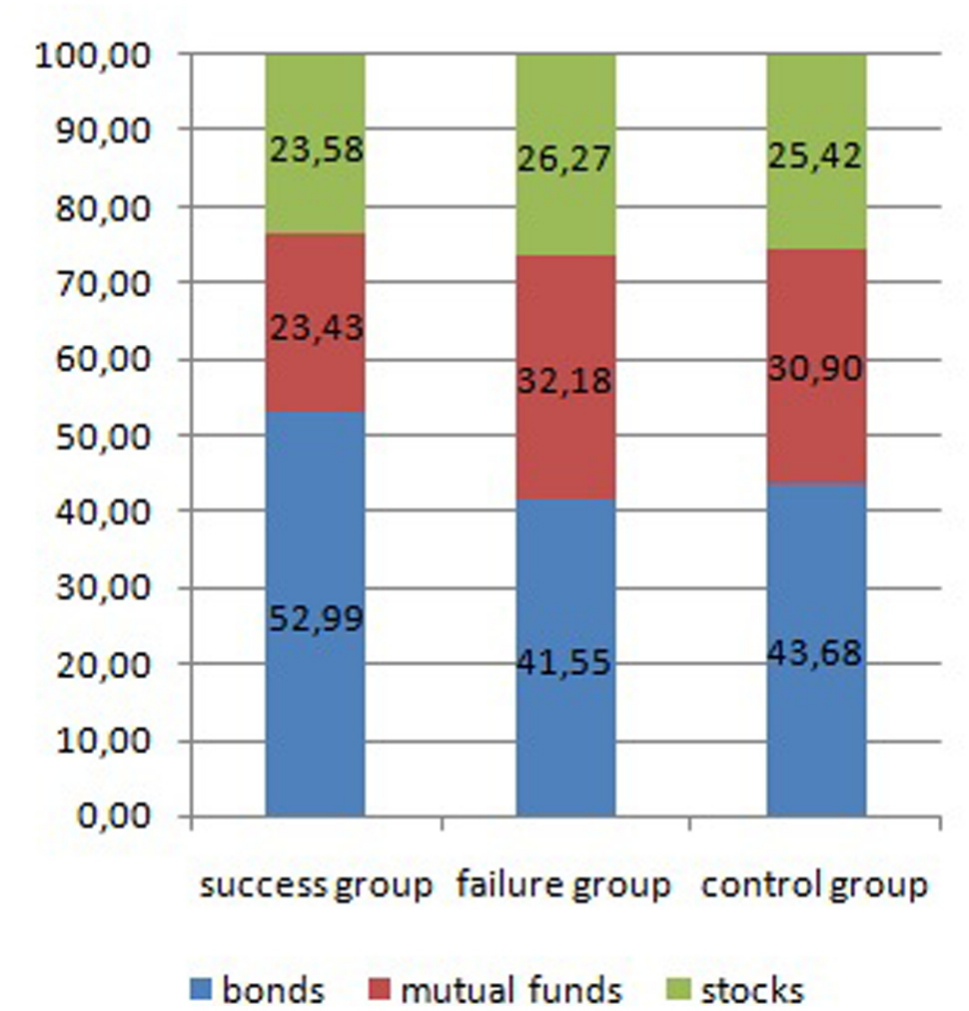
**Percentage of money assigned to invest in different forms of instruments in success, failure, and control groups – Study 2**.

To further verify the second research hypothesis, a 3 (experience: success, failure, control – between subjects IV) by 3 (form of investment: bonds, mutual funds, stocks – within subjects IV) mixed-design ANOVA, with a percentage of amount of money assigned by participants as a DV, was conducted. A significant interaction between experience and form of investment was obtained (*F*[4,522] = 2.643, *p* < 0.05, η^2^ = 0.020). Furthermore, the next ANOVA analysis revealed significant differences between all experimental conditions with regards to the propensity to invest in bonds (*F*[2,261] = 3.695, *p* < 0.05, η^2^ = 0.028) and mutual funds (*F*[2,261] = 3.370, *p* < 0.05, η^2^ = 0.025). The means and standard deviations of the three groups analyzed are reported in **Figure 4**. Further analysis using *t*-tests showed that participants from the success group were prone to spend more money on bonds and less on mutual funds than the failure (bonds: *t*[261] = 2.400, *p* < 0.05, *Cohen’s d* = 0.41, mutual funds: *t*[170] = 2.684, *p* < 0.05, *Cohen’s d* = 0.36) and control groups (bonds: *t*[261] = 2.073, *p* < 0.05, *Cohen’s d* = 0.30, mutual funds: *t*[161] = 2.040, *p* < 0.05, *Cohen’s d* = 0.31). No difference was observed between the three groups in terms of their tendency to invest in stocks (*F*[2,261] = 2.261, *p* = 0.29, η^2^ = 0.002).

## Discussion

The studies presented in this paper sought to examine the effects that the experience of success or failure in financial and non-financial tasks had on financial choices, including consumption, saving, investing and risky investment decisions.

Findings of the described research on consumption showed lower propensity to consume after a positive financial experience (but only when a small amount was allocated) but higher propensity to consume after a positive non-specific experience (but only when the large amount was allocated). The results did not show a role of experience of failure in explaining the propensity to consume and did not indicate any significant differences in propensity to consume between people who experienced success and failure. However, there is a possible explanation for these results. Mick and De Moss (1990) conducted a survey showing that personal accomplishments and achieved goals (in other words the experience of success), are important reasons why people reward themselves. Some examples of self-reward are material goods like sweets, new cloths or home electronics. KPMG survey in China showed that over 60 percent of participants bought themselves some luxury goods as a reward for their success (Debnam and Svinos, 2006). On the other hand, people who experience failure in some task can feel that their view of themselves is threatened and thus be motivated to engage in some compensatory behaviors to restore the status quo (Higgins, 1987). Many previous studies have indicated that consumption is one of the means willingly chosen by people to compensate for self-threats (i.e., Lee and Shrum, 2013; Rucker and Galinsky, 2013). These findings suggest that both experience of success and experience of failure may increase people’s propensity to consume which can be the explanation why there were no differences between success and failure groups observed in the current studies. The question is why there was no effect of failure on consumption and why the role of the success was inconsistent? Koch et al. (2014) postulated that different goods have a different motivational strength for an individual not only because they differ in terms of prices but also because they vary in terms of their value to the person. Compensatory consumption after negative experience aims to buy products that signal success in the specific domain of self-threat (Levav and Zhu, 2009; Lisjak et al., 2015). There are plenty of studies that confirm this statement in different domains of threat, e.g., intelligence (Kim and Rucker, 2012), power (Rucker and Galinsky, 2008) or social system (Cutright et al., 2011). Consumption after the experience of success, as a self-reward may not be related to the area in which the person succeeded (Mick and De Moss, 1990). Given these findings it is hypothesized that the role of the experience of success and failure on consumption may be more qualitative than quantitative, thus it is possible that the stronger effect may appear instead when we ask about the type of goods people are willing to buy, not the amount of money they are willing to spend on them. However, this is only a hypothesis that may be tested in a future study.

The findings from the reported studies showed that the experience of success and failure in a prior task influenced people’s propensity to save, invest and risky investment choices. Study 1 showed that the experience of success in financial tasks lowered the propensity to save and to choose safe investment instruments, but increased the propensity to invest (especially in stocks) and to create a risky investment portfolio. The experience of failure in financial tasks significantly increased safe investment preferences and the propensity to save, but lowered the general tendency to invest, to create a risky investment portfolio and to invest in stocks. The findings from Study 2, on the other hand, were exactly the opposite. People who experienced success in non-financial tasks preferred to save more, invest less, and create safer investment portfolios with a large amount of bonds. The failure group was less prone to save and more likely to invest and create risky investment portfolios. These results support the hypothesis that the role of the experience on financial choices may be different when it comes from specific (financial) than non-specific (non-financial) task.

The financial tasks after the priming of success or failure were the same in both studies, the only difference was the priming task. The experience of success or failure affects mood, regardless of the kind of task, as noted, for instance, by Isen and Simmonds (1978). In accordance with the mood maintenance theory people seek to maintain a good mood; they therefore do not want to jeopardize it by a potential loss on an investment (e.g., Leith and Baumeister, 1996). Consequently, people in a pleasant mood in both studies should prefer to save rather than invest; if they do invest, they should prefer safer instruments. Moreover, whatever the kind of task, the experience of success stimulates self-affirmation, while the experience of failure threatens self-esteem (Josephs et al., 1992). People prefer to reinforce their self-esteem and reduce threats to it (Josephs et al., 1992). Thus, people in both studies are supposed to prefer safer financial options after success, but may be more prone to risky investments after failure. These arguments could explain the results of Study 2 (experience in non-financial tasks), but the findings from Study 1 indicated the opposite. One possible explanation is that the experience of success in a preceding financial task (in contrast with experience in a non-financial task) may raise individuals’ belief in their financial self-efficacy. Furthermore, in accordance with the AIM (Forgas, 1995), the experience of success in a financial task creates a very fresh memory of success in a situation related to investing, that makes people more prone to risky choices. Prior experience in a financial task makes people also more familiar with the investment situation, so they may become less risk averse. The important role of familiarity on investment decisions was shown for example by Massa and Simonov (2006). Moreover, the experience of success can increase people’s tendency to overestimate the probability of success, and foster over-confidence in their own predictions; it can be expected that the effect is stronger in the case of experience in the specific task (in the context of subsequent decisions) than in terms of a non-specific task (e.g., Bandura and Cervone, 1986). Failure, however, creates the opposite effect. Participants in both studies were asked to create an investment portfolio, which is the investment task, for which, in real life, good results depend on the knowledge and skills of the individual. Thus, in accordance with previous studies (Au et al., 2003), people in a good mood are supposed to prefer a rather aggressive portfolio, while people in a bad mood safer ones. However, people in the first study were asked to make another investment choice a few minutes earlier (experimental manipulation). They were asked about stock selection, estimating their future value, based only on the share price charts. Thus, probably when decisions were made, participants in study one were more aware that the choice of financial instruments requires a broad knowledge. These arguments may explain the results obtained in the first study and their discrepancies with the results of the second study, but further studies are needed to verify those explanations.

In summary, the results of the reported studies suggest that the experience of success and failure in the preceding task affects financial choices, especially people’s propensity to save, invest and create a risky investment portfolio. Furthermore, how it affects the subsequent choices depends on whether the experience was in a specific task (in the context of financial choices) or in a non-specific one.

The studies presented in this paper partly fill a gap in the research on the role of experience in both financial and non-financial tasks preceding financial choices. Existing knowledge about the impact of the experience prior to financial choices was limited almost exclusively to individual risky choices. The reported studies complement the existing knowledge about the conclusions on the propensity to consume, save, invest and build risky investment portfolios. Research in previous studies mentioned the reasons for the ambiguous impact of experience on the risk preferences for the type of decisions (e.g., how they were important for the individual or whether the decision-making situation was ambitious and required knowledge and skills). The presented studies showed another way to explain these discrepancies, indicating the role of the specific circumstances in which the individual has experienced success or failure.

### Limitations and Future Studies

The studies have some possible limitations. The first is the methodology of the pilot studies of the experimental manipulations. The participants’ mood was measured only after the experience, not both prior and after it. Therefore clear-cut conclusions cannot be drawn. It would be worth checking mood changes after the manipulation again, taking into account the two measurements – before and after the experience.

The second limitation of the studies is the ratio of women to men among the participants in both studies. In each experimental condition (success, failure and control group) the ratio of women to men was the same in all three groups. Previous research has shown that women are more averse to risk than men (Hartog et al., 2002; Agnew et al., 2008); it is therefore important to replicate the studies with the participation of an equal number of men and women.

## Author Contributions

I am (KS) the only author of the manuscript. My role in creating the manual script included:

(1) Substantial contributions to the conception or design of the work; or the acquisition, analysis, or interpretation of data for the work; and
(2) Drafting the work or revising it critically for important intellectual content; and
(3) Final approval of the version to be published; and
(4) Agreement to be accountable for all aspects of the work in ensuring that questions related to the accuracy or integrity of any part of the work are appropriately investigated and resolved.

## Conflict of Interest Statement

The author declares that the research was conducted in the absence of any commercial or financial relationships that could be construed as a potential conflict of interest.

## Footnotes

1 The values of the small, medium and large amounts were established on the basis of a pilot study.

## Supplementary Material

Figure S1 **The tool used in the experimental manipulation in Study 2**.
