# Supplementary figures and images for: People’s Financial Choice Depends on their Previous Task Success or Failure
Source: Front Psychol. 2015 Nov 17;6:1730. doi: 10.3389/fpsyg.2015.01730 (PMC4646967; doi:10.3389/fpsyg.2015.01730)

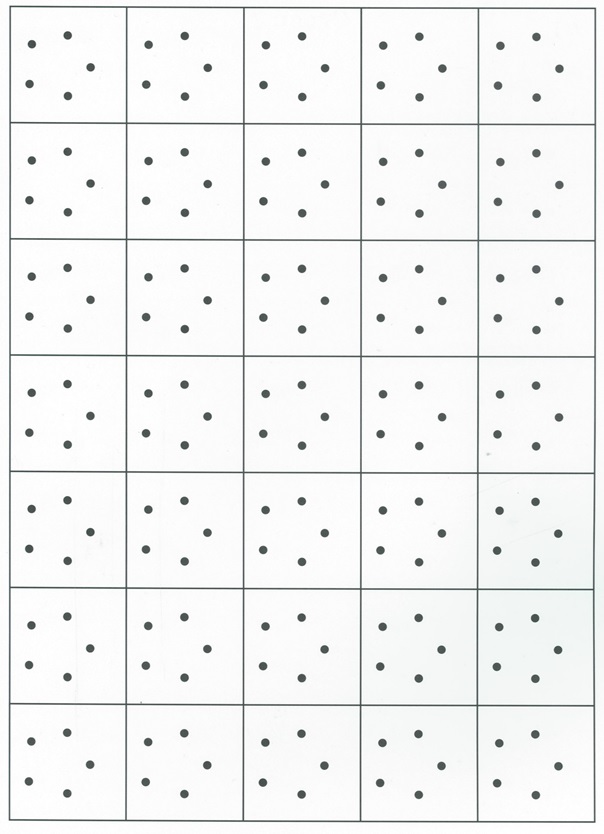

Supplement: Figure S1 — The tool used in the experimental manipulation in Study 2. [file Image_1.JPEG]
